# Suitability of existing definitions for drowning to meet the new definition: a scoping review 

**DOI:** 10.5249/jivr.v15i2.1816

**Published:** 2023-07

**Authors:** Ali Davoudi kiakalayeh, Reza Mohammadi, Shahrokh Yousefzade chabok, Sajad Davoudi kiakalayeh

**Affiliations:** ^*a*^ Department of Preventive and Social Medicine, School of Medicine, Guilan University of Medical Sciences, Rasht, Iran.; ^*b*^ Division of Family Medicine and Primary Care, Division of Social Medicine, Department of NVS, Karolinska Institutet, Stockholm, Sweden.; ^*c*^ Guilan Road Trauma Research Center, Guilan University of Medical Sciences, Rasht, Iran.

**Keywords:** Drowning, Scoping, Definition, Iran

## Abstract

**Background:**

Past studies have shown a lack of consensus on the definition and terminology of drowning among experts in the field and relevant organizations. There is a need for a new look at the definition of drowning to improve the understanding of drowning events.

**Methods:**

A literature search of seven electronic databases, including PubMed, EMBASE, CINHAL, MEDLINE, Sport Discus, and Social Sciences from 1960 to 2020 was conducted using the MESH search terms "drowning", "near-drowning", "submersion", and "immersion". Cochrane databases were also searched for systematic reviews The items were searched in all fields of publication, including title, abstract, and keyword.

**Results:**

The search identified approximately 2500 articles, 230 of which were reviewed. The inclusion criteria were applied to the full text of 230 articles, and 25 articles addressing the different definitions of drowning were assessed. They were reviewed critically by authors using a standard review form. The search identified that there were at least 20 different outcome measures for drowning incidents reported. Definitions of drowning in the literature were found for the following terms: dry versus wet drowning, secondary drowning, drowned and near-drowned, drowning without aspiration and drowning with aspiration, near drowning without aspiration or with aspiration, active drowning, passive drowning, silent drowning, witnessed and unwitnessed, immersion, submersion, death certificate records drowning, unintentional submersion, road traffic injury leading to passenger vehicle drowning, drowning, near drowning, salt or freshwater drowning, and cold water drowning.

**Conclusions:**

In the literature, a lack of consensus was observed but the following terms should not be abandoned; "Non-fatal drowning" which is used to describe death following rescue and life with at least 24 hours of in-hospital survival and the development of one or more complications and "Fatal drowning" which implies death occurring at the scene or 24 h of a submersion incident.

## Introduction

Recreational aquatic activity is usually associated with immersion/ drowning in Iran and many developing countries.^1^ According to recent studies, drowning accounts for more than 1500 annual deaths in Iran, with a fatal drowning rate of 2.9 per 100,000 population in two provinces.^2^ There are many definitions of drowning that can affect epidemiological data collection. In response to these problems, a simple but comprehensive definition of drowning was explored during the WCD (World Congress on Drowning) meeting in 2002; “Drowning is the process of experiencing respiratory impairment due to submersion or immersion in a liquid”.^3^ Drowning is an event that begins with immersion in liquid and progresses to respiratory system dysfunction, ultimately leading to death in the absence of rescue and relief.^4-7^ Drowning outcomes are classified as fatal, non-fatal and morbidity. Therefore, a victim can be rescued at phases of event. This definition creates a tool basis for next injury surveillance studies in the worldwide. However, a wider definition is uses in the literatures ranging from no morbidity to fatal drowning. Efforts to address leading causes associated with drowning, such as inadequate supervision, lack of barriers, and lack of swimming ability, can help prevent drowning fatalities and improve public health outcomes. Emergency medical services play a main role in the resuscitation and monitoring of drowning patients. To create a drowning prevention program, researchers need to have a common language regarding drowning to make the concept comparable among researchers from different backgrounds. A previous study in 2005 described 33 different definitions for drowning from 1966 to 2002,^8^ demonstrating a gap of common definitions as a key problem in literature reports. Therefore, to have high-quality data for creating a drowning prevention program, all relevant cases of drowning, both fatal and non-fatal, should be counted because they can have an unknown impact on public health. To improve drowning surveillance, this study aimed to systematically review the all manuscript for the words used for describing drowning events and to alter the existing definitions to meet the new definition. 

## Materials and Methods

The study is extracted information from the Iran National Registry of Drowning (INRD), which is the national statistical database of Iran for registering drowning cases. The researchers conducted a literature search of seven electronic databases, including PubMed, EMBASE, CINHAL, MEDLINE, Sport Discus, and Social Sciences, from 1960 to 2020 using the search words drowning, drowning prevention, near-drowning, submersion, and immersion. Cochrane databases were assessed for systematic reviews. These main keywords were targeted in the studies related to drowning, such as title, abstract, and keyword. Any relevant article with a definition of drowning/submersion was included and explored. Exclusion criteria including drowning because of suicide or homicide, and other intentional drowning. However, this study focuses to explore studies on unintentional fatal drowning and non-fatal drowning.

## Results

The search result finds out approximately 2500 manuscripts, of which 230 were observed. Most articles were observational studies and randomized controlled trials did not refer to the drowning definition. The inclusion main keywords were uses to the full texts of 230 manuscripts, and 25 papers addressing the different definitions of drowning were evaluated. The 25 papers were assessed completely by authors using a standard review guideline form. More than 20 different outcome measures for the reported drowning/submersion events were found. The literature review identified different definitions of drowning, including” drowning without aspiration, drowning with aspiration, near drowning without aspiration, near drowning with aspiration, and dry drowning”. The words wet and dry drowning should no longer be used. Any submersion or immersion events without sign of respiratory dysfunction (aspiration) should be assessed a water rescue and not a drowning.

**Definitions of submersion and drowning in the studies were as follows:**

**1- “Submersion”: **In submersion process, the victim is entered in water and the face and airway are covered by water.^9^

**2- “Drowning”:**Death due to suffocation within 24 h of submersion process in a water medium.^10^

**3- “Near Drowning”:**After at least 24 hours of suffocation due to submersion in the water, the victims is survived.^10^

**4- “Salt or freshwater drowning”:**Severe alveolar-arterial dysfunction of oxygen following liquid aspiration.^11^

**5- “Secondary drowning”: **Refer to a process that can be caused by an unrelated event such as seizures in children, cervical spinal cord injury, and heart attack in the elderly, resulting in submersion/drowning.^12^

**6- “Drowning”: **Submersion injury resulting in death.^13^

**7- “Dry drowning”: **A laryngeal spasm with little aspiration of water.^14^

**8- “Cold water drowning”: **Cold water drowning can occur when a victim falls into cold water during the autumn, and winter seasons and four phases of cold-water drowning are happened as possible.^15^

**9- “Near drowning without aspiration”: **Refers to survive following asphyxia due to submersion in water.^16^

**10- “Freshwater drowning”: **Suffocation in the freshwater or seawater following by submersion and resulting death.^17^

**11-“Immersion” **is when the victim is entered in water while the face and airway are not immersed yet.^18^

**12- “Drowning without aspiration”: **Death due to respiratory dysfunction and asphyxia while submerged in liquid.^19^

**13- “Drowning with aspiration”: **Death due to combined effects of laryngospasm and cerebral hypoxia.^20^

**14- “Road traffic injury leading to passenger vehicle drowning”.**^21^

**15- “Resulting due to Unintentional submersion injury”.**^22^

**16- “Death during 24 hour of a submersion events”.**^23^

**17- “Wet drowning”: **Aspirating liquid into the lungs.^23^

**18- “Drowning as a cause of death in the death certificate”.**^24^

**19- “Drowned”: **Refers to a person who died from cerebral hypoxia after aspiration of liquid while submerged.^25^

**20- “Passive drowning and silent drowning”: **Finding the victim standstill in the liquid with no one witnessing the victim's entrance into the liquid.^25^

**21-“Near-drowned”: **Which describes victims who survive at least temporarily, and subsequently die from drowning.^26^

**22- “Active drowning”: **An event is a person who see in which the victim is making some movement.^26,27^

**23- “Near drowning with aspiration”: **To survive the following initial response to aspiration of liquid while submerged.^28^

**24- “Witnessed”: **Refer to onset of drowning episode is saw by someone.^29,30^

**25- “Unwitnessed”: **No one saw the onset of drowning episode a victim in the water.^29,30^

## Discussion

In this context, the literature review identified 25 different definitions of drowning, indicating a gap of a corrected definition of drowning/submersion and absence of standard on outcome measures. However, efforts have been made to improve a standardized drowning/submersion terminology using a consensus-based approach, which allow consistency in nomenclature and data reporting on drowning. To ensure a consistent approach to terminology related to drowning/submersion, many instructions have minimum agreement of definitions. Improved and more comprehensive reporting is a priority in identifying the drowning/submersion problem and providing effective prevention interventions. Drowning is a preventable health system problem worldwide, with a number 500,000 deaths in each year.^31^ However, in many developing countries like Iran, this number is probably against the actual figures due to the misunderstanding of the definition of drowning, resulting in underreporting of actual cases.^32^ Improved and more comprehensive reporting is a priority in identifying the drowning problem and improving effective prevention interventions. Efforts to address leading causes associated with drowning, such as inadequate supervision, lack of barriers, and lack of swimming ability, can help prevent drowning fatalities and improve public health outcomes. The scientific literature has shown that there is much more variability in definitions in the case of near-drowning than in drowning cases.^33-34^ Using the term near-drowning has created an confusing meaning of the drowning/submersion process, and when the term is translated from English into other languages, the meaning can be confusing to researchers. The study recommended that words in the definition of near-drowning should be eliminated when exploring drowning/submersion in the scientific literature. 

According the WCD report in 2002, a new language in defining drowning was released , which includes the following definitions:^3^ drowning/submersion without or with morbidity ; drowning without morbidity is defined when the victim is alive with no cerebral complication following the submersion/immersion episode; drowning with morbidity is defined when the victim is survived with cerebral side effect following the submersion/immersion episode, including moderately or severely disabled, comatose, and brain death; and fatal drowning is defined when the victim has succumbed to a submersion/immersion episode. The term drowning/submersion is based on validated measures outcomes of cerebral complications. Agreed terminology is essential to explore the problem and to allow effective comparisons of drowning trends. The recommended definition of drowning should be based on the pre-event, event, and post-event drowning processes. For the pre-event phase of the drowning process, the definition should focus on people at risk, and the drowning definition should be based on victims’ factors associated with increased risk of death, including being male, having a seizure disorder, and use of alcohol. For the event phase of the drowning process, the drowning definition should be based on the prolonged duration of submersion and failure to receive standard CPR. In the post-event drowning process, the definition of drowning should consider morbidity, which can be quantified at long-term follow-up after hospital discharge, best describing the outcome. The main reason for this classification is that on-scene drowning data in developing countries are being underreported due to confusing definitions of drowning. Most rescue and relief begin at the scene of the drowning/submersion, especially in the Sea area, before victims are transported to a hospital when they recover at the scene. Therefore, drowning definition can be classified as fatal and non-fatal drowning. The Non-fatal Drowning Categorization Framework (NDCF) proposed by WHO categorizes non-fatal drowning along two dimensions: the severity of respiratory impairment and the duration of hospitalization. The literature recommends adding all different studies on drowning that are published in non-peer-reviewed journals or non-English articles. However, it is important to ensure that these studies meet the same rigorous standards as peer-reviewed studies.

## Conclusion

To have an effective drowning prevention program, the following terms should not be abandoned. This study recommends both definition for drowning: non-fatal drowning describes death following rescue and life with at least 24 hours of in-hospital survival and the development of one or more cerebral side effects, and fatal drowning refers to death occurring at the scene or 24 hours after a submersion incident. 
